# Effect of Sand Fines Concentration on the Erosion-Corrosion Mechanism of Carbon Steel 90° Elbow Pipe in Slug Flow

**DOI:** 10.3390/ma13204601

**Published:** 2020-10-16

**Authors:** Rehan Khan, Hamdan H. Ya, William Pao, Mohd Amin Abd Majid, Tauseef Ahmed, Amir Ahmad, Mohammad Azad Alam, M. Azeem, Hassan Iftikhar

**Affiliations:** 1 Mechanical Engineering Department, Universiti Teknologi PETRONAS, Perak 32610, Malaysia; william.pao@utp.edu.my (W.P.); mamin_amajid@utp.edu.my (M.A.A.M.); tauseef_17007229@utp.edu.my (T.A.); mohammad_18000664@utp.edu.my (M.A.A.); mohammad_18000380@utp.edu.my (M.A.); 2 Group Technical Solution PETRONAS, Menara Dayabumi, Kuala Lumpur 50050, Malaysia; amir_ahmad@petronas.com; 3 Department of Mechanical Engineering, Lappeenranta University of Technology, Yliopistonkatu 34, 53850 Lappeenranta, Finland; Hassan.iftikhar@student.lut.fi

**Keywords:** erosion-corrosion, concentration, slug flow, sand fines, elbow

## Abstract

Erosion-corrosion of elbow configurations has recently been a momentous concern in hydrocarbon processing and transportation industries. The carbon steel 90° elbows are susceptible to the erosion-corrosion during the multiphase flow, peculiarly for erosive slug flows. This paper studies the erosion-corrosion performance of 90° elbows at slug flow conditions for impact with 2, 5, and 10 wt.% sand fines concentrations on AISI 1018 carbon steel exploiting quantitative and qualitative analyses. The worn surface analyses were effectuated by using laser confocal and scanning electron microscopy. The experiment was conducted under air and water slug flow containing sand fines of 50 µm average size circulated in the closed flow loop. The results manifest that with the increase of concentration level, the erosion-corrosion magnitude increases remarkably. Sand fines instigate the development of perforation sites in the form of circular, elongated, and coalescence pits at the elbow downstream and the corrosion attack is much more obvious with the increase of sand fines concentration. Another congruent finding is that cutting and pitting corrosion as the primitive causes of material degradation, the 10 wt.% sand fines concentration in carrier phase increases the erosion-corrosion rate of carbon steel up to 93% relative to the 2 wt.% sand fines concentration in slug flow.

## 1. Introduction

It is well-known that erosion-corrosion is one of the ubiquitous deterioration modes of piping components [1]. Erosion-corrosion could cause significant degradation of pipelines in hydrocarbon production fields and result in an unpredictable failure of the piping components and operating process [2,3]. Sand particles are often transported in pipelines from reservoirs in multiphase flow configurations during the production process and induce erosive wear in piping components [4,5]. Elbow pipes are extensively used to redirect the flow in hydrocarbon and mineral processing industries, the erosive wear in a 90° elbow pipe configuration conceivably 50-times higher than that in straight pipe configurations under multiphase flow [6].

Multiphase flow in pipelines is characterized by the distribution of two or more phases into heterogeneous flow regimes as they flow concomitantly in a pipe [7]. The phases that can be contemporary in a multiphase flow are solids, liquids, and gases. A physical understanding of the flow characteristics with more than one phase is much more complex than single-phase as the phases are distributed in different configurations. In horizontal–horizontal orientation, the flow patterns are generally categorized as plug, slug, low hold up wavy, and annular flow [8].

The slug flow regime is commonly observed for sand transportation in liquid and gas phases. Due to the disparity in carrier phase densities, the gas bubbles inhabit the upper portion of the pipe and are segregated from the continuous liquid phase adjacent to the lower portion of the horizontally oriented pipe [9]. The intact pipe cross-sectional area can be occupied by slugs of liquid isolated by regions of the gaseous phase. In the erosive slug flow pattern, the slug body is the primary source of erosion because the erodents are transported solely by the liquid phase and the slug body has the highest liquid holdup. The majority of the entrained sand travels in the liquid of the slug body and induces erosion-corrosion damage in pipelines [10].

In literature, the large number of experimental investigations on erosive wear has been performed on the domain of multiphase flow in the last decades such as studies done by Sedrez et al. [11], Zahedi et al. [12], Vieira et al. [13], Parsi et al. [14], and Owen et al. [15]. However, experimental investigations, with all the recent developments, were not triumphant in precipitating results with acceptable accuracy in multiphase flow ascribed to the involvement of the highly turbulent liquid and solid particle interactions [16]. Postlethwaite and Nesic [17] mentioned that the turbulent flow, pressure impact, solid particle impaction, and suspended liquid droplets impaction are the sources of mechanical forces that cause erosion-corrosion in multiphase flow. Postlethwaite [18] observed that the wear rate of metals is enhanced by corrosion during erosion-corrosion through roughening of the target surface which degrades the exposed area. Keating and Nesic [19] used a combined Eulerian and Lagrangian computational methods to predict erosion-corrosion in a square-sectioned U-bend.Previous research on solid particle erosion showed that the erodent impact angle strongly influences the extent of erosion [20,21,22,23]. In steel material, the material removal rate is the maximum between 15°–30° impact angles and cutting, and ploughing is the dominant degradation mechanism that causes the inflated erosion level. Oka et al. [24] conducted an erosion test which concluded that the particle impact angle function is not reliant on erodent velocity.

A more elaborate experiment was conducted for an elbow configuration by Elemuren et al. [25]. They measured the surface roughness parameters of an elbow and the surface topographies were extracted from the upstream, middle, and downstream sections. The results reveal that the material removal inside elbows originated by abrasion/sliding mechanism as slurry flow to downstream. Recently, Cao et al. [26] performed an experiment to quantify the erosion rate in 90° elbows for slug flow with sand particles. They found the elbow outer wall adjacent to exit was more susceptible to erosive wear than other positions.

Peng and Cao [27] investigated elbow erosion for multiphase flow and found that maximum erosion occurs adjacent to outlet due to the cumulative effect of sliding and direct impaction. Uzi et al. [28] scrutinized the particle wall interaction of the elbow pipe and concluded that the maximum particle wall collation was at 53° along the curvature despite the particle size. Mansouri et al. [29] investigated numerically and experimentally the erosive wear of elbows configurations for quartz particles. Rawat et al. [30] found that an impact angle of 45° causes maximum erosion wears in fly ash slurries. Al-lababidi et al. [31] experimented to study sand transported in straight pipes for slug conditions and observed the liquid film zone escalation with inflation in the gas phase velocity.

At present, there are limited experimental studies available on erosive wear of 90° elbows concomitant to sand fines, especially when they are entrained in the slug flow. To avoid the progression of larger sand particles in the main production line the sieve mesh was extensively deployed at the entrance section, however, the standard sieve mesh cannot restrain sand fines progression in a fluid phase [32]. Furthermore, the sand concentration is a critical parameter for assessing erosion induced damage as well as it also has a significant effect on the erosive wear mechanism of the target material [33,34]. However, in all the available studies of elbow configurations, only a single level of particle concentration was used to simulate the amount of erodent in carrier phase which does not provide the whole detail about the influence of sand concentration on erosion-corrosion rate and extent of erosion. Moreover, sand fines transport at lower flow velocities and low concentration levels can cause the formation of a deposition layer that enhances erosion and corrosion problems [16,35].

In this work, an experimental procedure is effectuated to assess and quantify the erosion-corrosion of 90° elbows in slug flow conditions. The procedure is adapted for the horizontal oriented AISI 1018 steel elbows with a 50.8 mm diameter in water and air carrier phases. Also, sand fines of average size 50 µm and concentration were about 2%, 5%, and 10% by weight used to evaluate erosion-corrosion damage. The complete surface morphologies and microscopic images of the test elbows exit section are obtained to scrutinize the sand fines erosion-corrosion mechanism. The degradation rate of the 90° elbow configuration is quantified by using the mass loss information. The erosion profiles on elbows internal surface are achieved through the paint modeling approach and analyzed qualitatively for different sand fines concentrations.

## 2. Experimental Methods and Materials

**Figure 1:**
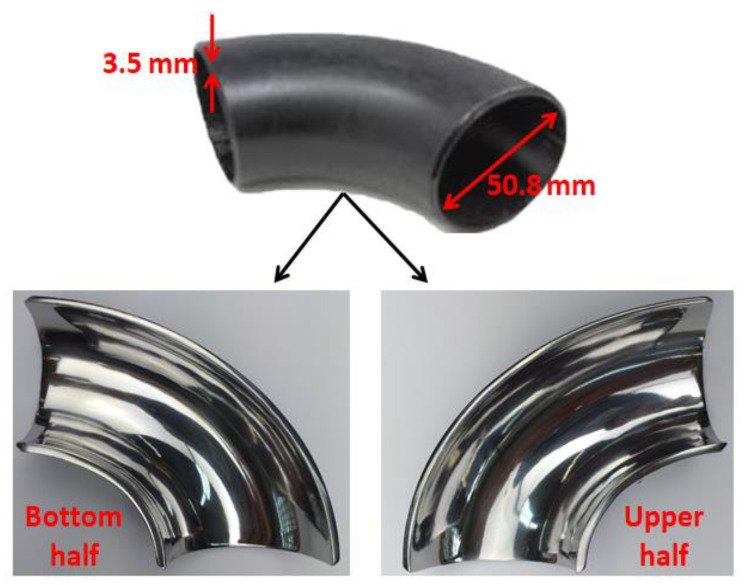
Axially cut sections of a fine polished 90° elbow.

**Figure 2:**
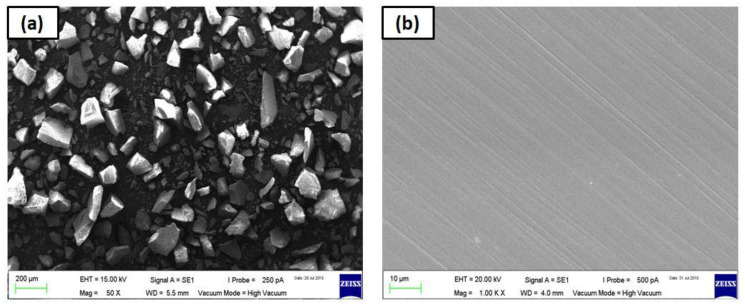
Microscopic images of: (**a**) silica sand, and (**b**) polished 1018 carbon steel (CS).

The 90° elbow test specimens used are of carbon steel having 98% iron and 0.2% carbon as outlined in Table 1. The density of the specimen material is 7870 kg/m^3^ and VHN (Vicker hardness) of the test elbow is 168 on the Vickers scale. The 90° elbow test specimen is 3.5 mm thick with 50.8 mm internal diameter and axially cut in the bottom half and an upper half section with wire electro-discharge machining (WEDM) process as illustrated in Figure 1. These elbow specimens’ internal surfaces are reduced to low surface roughness by employing a variable speed pneumatic angle grinding machine with a flap wheel grinding tool. For each experiment, a new test elbow is used with identical roughness levels to ensure cognate conditions for each test. It is also ensured that the internal surface of the specimens is not exposed to the moisture content to avoid corrosion reactions at the internal surface before the test. In addition to polished specimen, separate test specimens were prepared for paint modeling experiment. The paint modeling detailed procedure and principle were described previously [8]. The microscopic image of the erodent and test material is presented in Figure 2.

The dispersed phase used for the experiments is the sand fines obtained from LSK enterprises in Ipoh, Malaysia. The chemical compositions of the dispersed phase are listed in Table 2. The average erodent size of 50 ± 2 µm is determined using the laser scattering particle size distribution analysis. A 3-D non-contact profiling technique described in previous work [8,36] was applied to obtain surface topography data of the worn elbow section. To be able to evaluate the changes in the pit development mechanism after erosion-corrosion tests, a scanning electron microscopic technique was introduced in this work.

### Multiphase Flow Loop Apparatus and Medium

**Figure 3:**
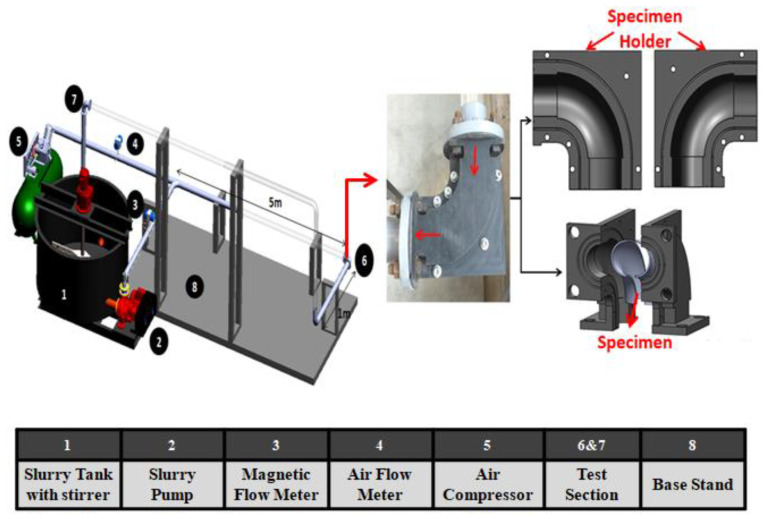
Experimental setup used for the present investigation.

Figure 3 illustrates the experimental apparatus employed for testing of the erosion-corrosion of 90° elbow configurations in slug flow. The experimental flow loop equipped with a slurry tank, air compressor, slurry pump, test section, magnetic flow meter, and air rotameter. The first step of effectuating slug flow erosion experiments was to load the slurry tank with tap water without abrasive particles. Afterward, the control valves installed in the air compressor were regulated to maintain the required air flow rate in the flow loop.

**Figure 4:**
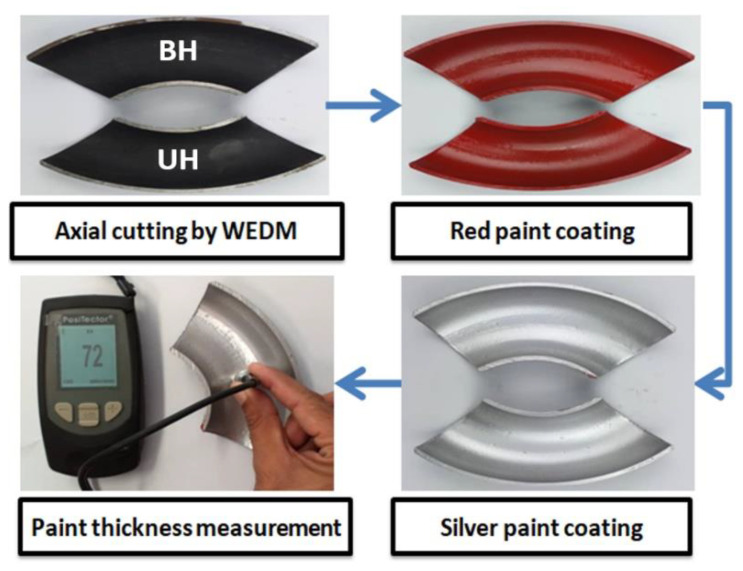
Multilayer paint modeling (MPM) sample preparation stages.

In all cases, the air was injected in the flow loop and subsequently, the variable speed slurry pump was turned on to transport the liquid phase into the flow loop. At this point, the liquid flow velocity was recorded using a magnetic flowmeter installed adjacent to the pump outlet. After attaining the preferred carrier phase flow rates, the fine sand particle was pre-pended in the mixing tank. A slurry stirrer was pitched into to avoid settling of the fine sand particles. Air flowing inside the loop was mixed with the slurry mixture and generates liquid-gas-solid flow in a pipeline and erodes the tested elbows at station 6 as shown in Figure 3. Details about the test section with the technique of mounting and sealing of the bottom half (BH) and upper half (UH) can be found in our previous published work [8,36]. Besides the erosion-corrosion rate quantification, erosion distribution mapping was conducted using a multiplayer paint modeling approach as shown in Figure 4. Experiments are performed for 1 h for multilayer paint modeling and 10 h for liquid-solid-gas so that a measurable weight loss was acquired.

**Figure 5:**
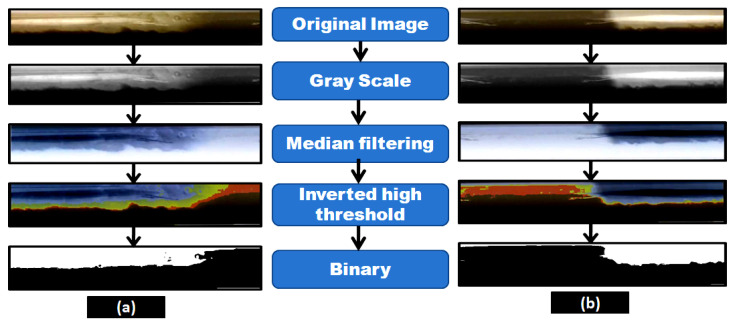
Images of slug flow: (**a**) bubble nose and (**b**) bubble tail.

After the experiment, the eroded specimens were washed with ethanol and dried with a heat gun and placed in desiccators to avoid moisture exposure. Mass loss was measured after achieving a steady-state condition using a precision electronic balance, transforming into kg/s·m^2^ to obtain erosion-corrosion rate. The slug flow regime visualization had been made through transparent acrylic pipe sections during the experiment for liquid superficial velocity 0.5 m/s and gas superficial velocity of 2.5 m/s based on Madhane et al. [37] flow pattern map. A slow-motion digital video camera has been employed to capture the slug flow patterns. The recorded original images as shown in Figure 5 was taken by the digital camera, and in the first step, the originally captured image was converted to the grayscale image. Afterward, the median filtering technique was employed to remove noise present in an image and transformed into HSI (hue, saturation, and intensity) inverted color space with a high threshold, where the gas-liquid interface in the image can be readily distinguished. Then, the resulted image is transformed into a binary image to visualize, bubble tail, and nose image of the slug flow regime as shown in Figure 5.

## 3. Results and Discussion

### 3.1. Multilayer Paint Modeling (MPM)

**Figure 6:**
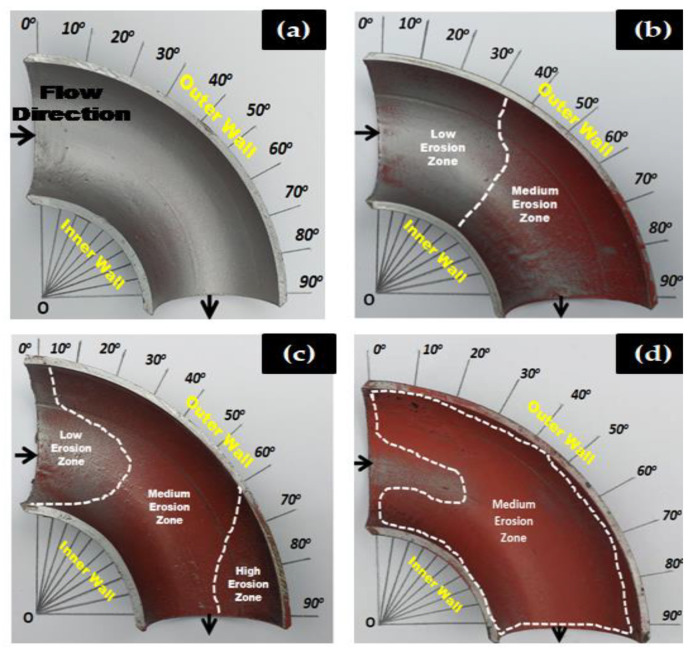
Paint erosion pattern for slug flow with *V*s_L_ = 0.5 m/s and *V*s_G_ = 2.5 m/s in a 90° horizontal-horizontal elbow bottom section. (**a**) Without sand [8], (**b**) 2 wt.% sand fines [8], (**c**) 5 wt.% sand fines [36], and (**d**) 10 wt.% sand fines.

**Figure 7:**
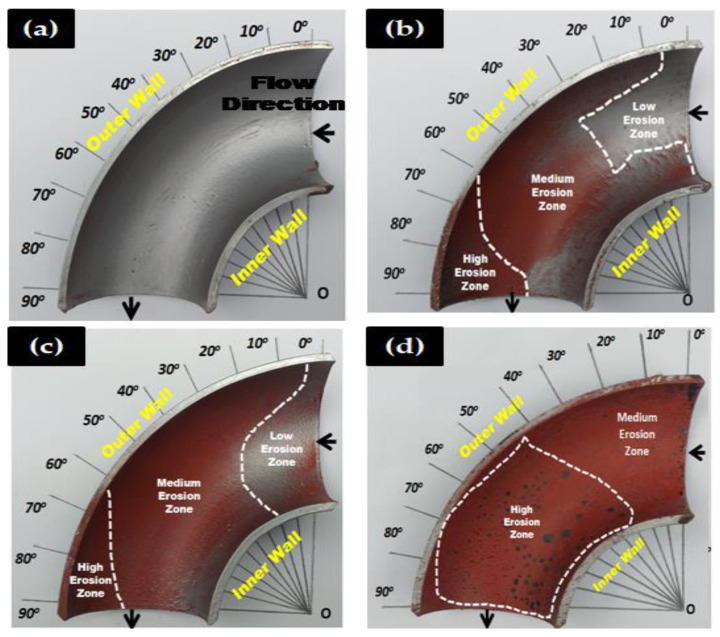
Paint erosion pattern for slug flow with *V*s_L_ = 0.5 m/s and *V*s_G_ = 2.5 m/s in a 90° horizontal-horizontal elbow upper section. (**a**) Without sand [8], (**b**) 2 wt.% sand fines [8], (**c**) 5 wt.% sand fines [36], and (**d**) 10 wt.% sand fines.

To compare the effect of sand fines concentration on erosion distribution, the MPM experiment was performed with 2, 5, and 10 wt.% sand fines at slug flow for air superficial velocity of 2.5 m/s and water superficial velocity of 0.5 m/s for 1 h of flow time. In paint erosion study, the inner surface of the elbow was painted with two different paint layers (Figure 6) and susceptible to sand fines impaction which evoked the paint layer to be eroded, and erosion patterns could readily distinguish. The paint erosion pattern caused by slug flow without sand fines was observed (Figure 6a and Figure 7a); however, the hydrodynamics of slug flow did not originate a paint erosion marks as shown in Figure 6 and Figure 7.

Figure 6 and Figure 7 give a closer look at the behavior of an extent of erosion at high sand fines concentration, as it approaches the 90° elbow. At 10 wt.% sand fines concentration, maximum particle wall impaction are seen on BH and UH section, the cumulative impact of erosive particles and air flowing on the upper section generate high erosion zone at elbow top and water flowing in the bottom of the pipe causes medium erosion at the bottom wall. Another interesting observation from the erosion pattern of paint indicates the inhabitance of black marks on the UH section (Figure 7d) was evidence of high particle concentration in the gas core.

Two other observations can be made here. First, the changing sand fines concentration significantly alters erosion location and extent of erosion. At 10 wt.% sand fines concentration, maximum paint erosion location were found between 30° to 90° adjacent to the outlet. In contrast, 2 wt.% and 5 wt.% concentration level, the maximum impaction location was relatively within 60° and 90° on the outer curvature of the elbow. The second observation is the circular eroded paint pattern between 30° and 90° in 10 wt.% sand fines, which is ascribable to the high particle–wall interactions in the upper section. To summarize, the results indicate the increase of sand fines concentration in the carrier phase significantly enhances erosion induced damage. The MPM results indicated the outer wall of the 90° elbow towards downstream is more susceptible to erosive wear and consistent with the obtained results of X. Cao et al. [26].

### 3.2. Surface Topographies and SEM Analysis

**Figure 8:**
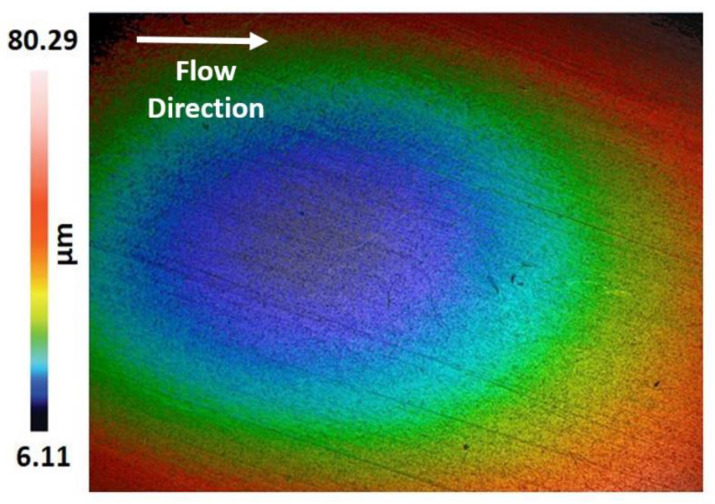
Confocal micrograph of 90° elbow exit section for slug flow without sand particles.

**Figure 9:**
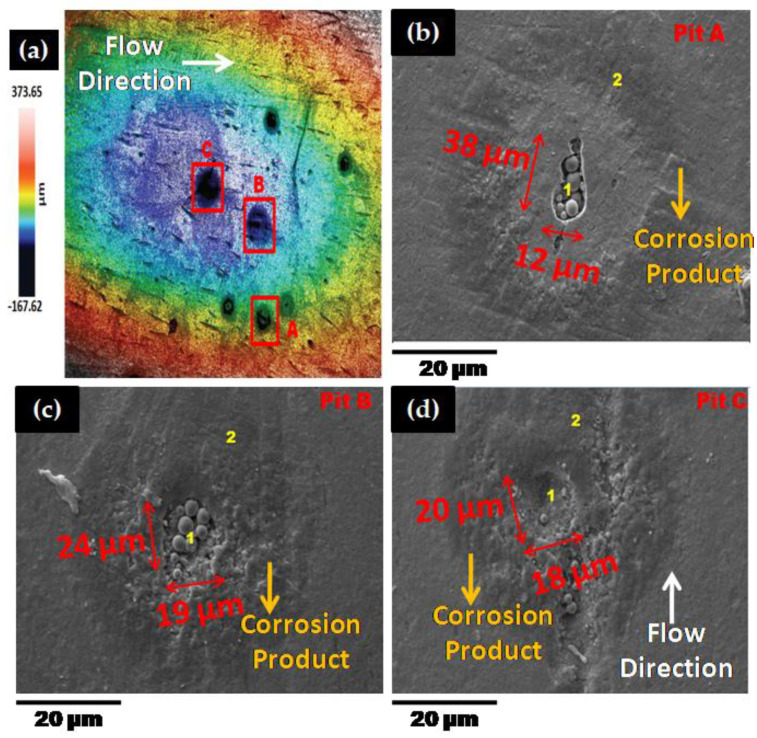
Micrographs of 90° elbow exit section for 2 wt.% sand fines concentration (**a**) confocal and (**b**–**d**) SEM inside pit (1) and outside pit (2).

**Figure 10:**
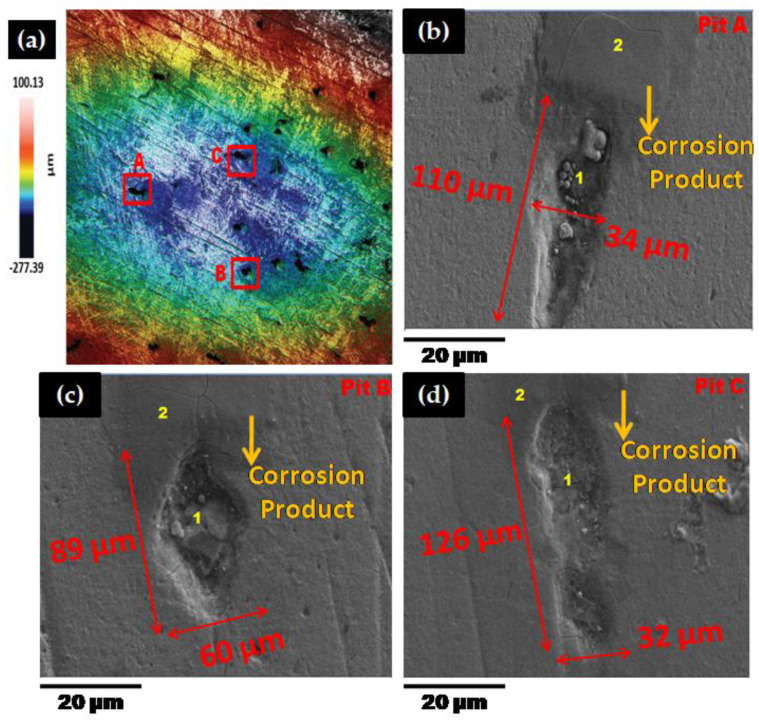
Micrographs of 90° elbow exit section for 5 wt.% sand fines concentration (**a**) confocal (**b**–**d**) SEM inside pit (1) and outside pit (2).

**Figure 11:**
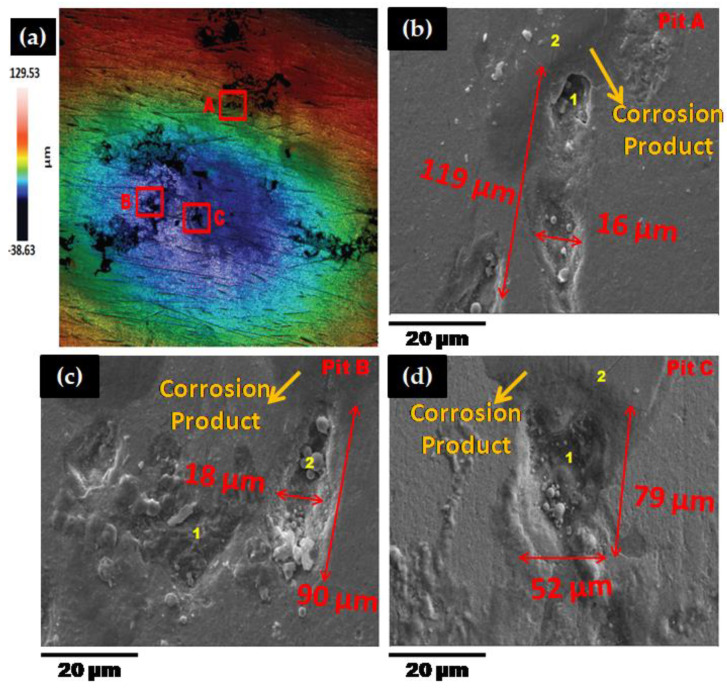
Micrographs of 90° elbow exit section for 10 wt.% sand fines concentration: (**a**) confocal and (**b**–**d**) SEM inside pit (1) and outside pit (2).

In this section, a laser confocal (Sensofar, Barcelona, Spain) and scanning electron microscopy (SEM, EVO LS 15, Carl Zeiss, Oberkochen, Germany) results were presented to evaluate the mechanistic development of erosion-corrosion zones on the 1018 CS 90° elbow specimen internal surfaces. The microscopic analysis was performed in the exit section of 90° elbow based on the findings of our previous research [36] that the exit section was more susceptible to erosion-corrosion than other positions in slug flow. The laser scanning confocal microscopy was performed at the exit section of the elbow samples for a scanning area of 3508.8 × 2641.9 µm^2^ to observe erosion-corrosion damage of carbon steel for different sand fines concentration levels in slug flow. Figure 8 shows the confocal micrograph of the exit section of 90° elbow after operating under slug flow conditions without an abrasive medium for 10 h in a multiphase flow loop. By employing the experimental flow loop depicted in Section 2, pitting morphologies were obtained after 10 h of the test with sand fines, as shown in Figure 9, Figure 10 and Figure 11.

The pits can be discerned on the 90° elbow surface after 10 h of erosive slug exposure and more pits were perceived on the surface of 90° elbow tested for 5 wt.% sand fines concentration compared to 2 wt.% sand fines concentration (Figure 9). With increasing sand fines concentration, in carrier phases, more pits detected on the elbow exit section and the small craters grew to larger elongated pits. Based on Figure 9 and Figure 10, with increasing the sand fines concentration (2 wt.% → 5 wt.%), the pitting corrosion morphology changes significantly from circular pits to elongated pits, and 10 wt.% sand fines concentration in slug flow resulted in the largest cumulative number of coalescence pits.

The number of pits in 2 wt.% sand fines concentration was fewer, which may be due to the less particle wall impaction and low particle loading, leading to fewer pitting sensitive sites. Nevertheless, it should be clear that fewer pits do not signify smaller pits or greater corrosion resilience. The size of the perforation sites is more important for the assessment of erosion-corrosion performance for multiphase flow conditions. It can also be perceived from the pit morphology in Figure 10 and Figure 11 that the length of the pits progressed to almost 126 μm after 10 h of the test with 5 wt.% sand fines concentration and 119 μm with 10 wt.% sand fines concentration, indicating serious localized erosion-corrosion damage in the 90° elbow exit section.

**Figure 12:**
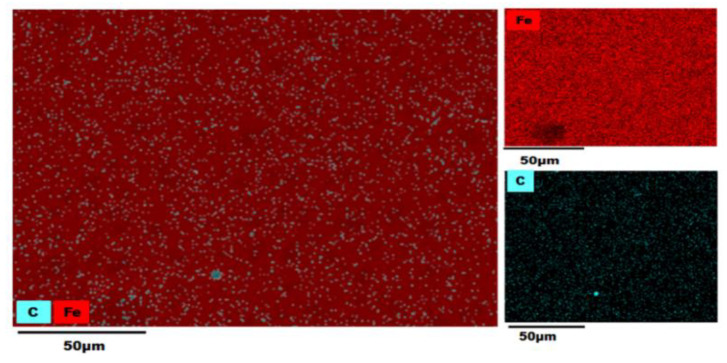
Energy-Dispersive X-ray Spectroscopy (EDS) map of a 90° elbow exit surface before the test.

**Figure 13:**
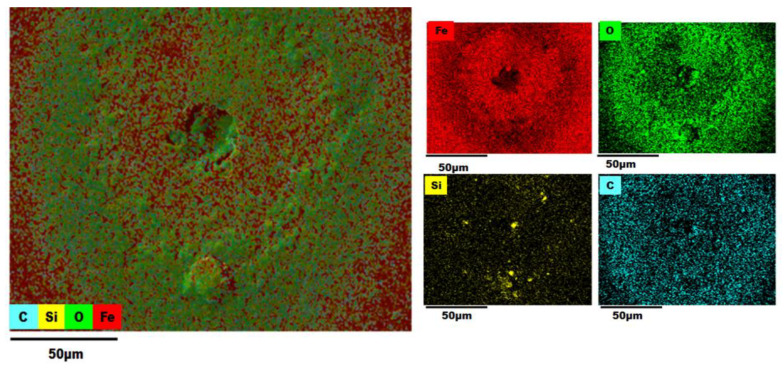
EDS map of a 90° elbow exit surface after the test for 2 wt.% sand fines concentration [8].

**Figure 14:**
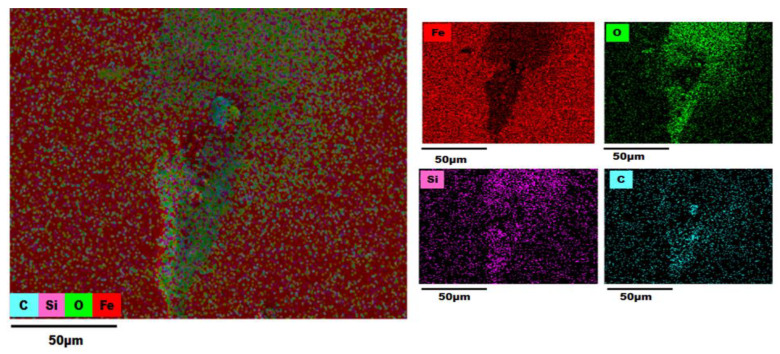
EDS map of a 90° elbow exit surface after the test for 5 wt.% sand fines concentration.

**Figure 15:**
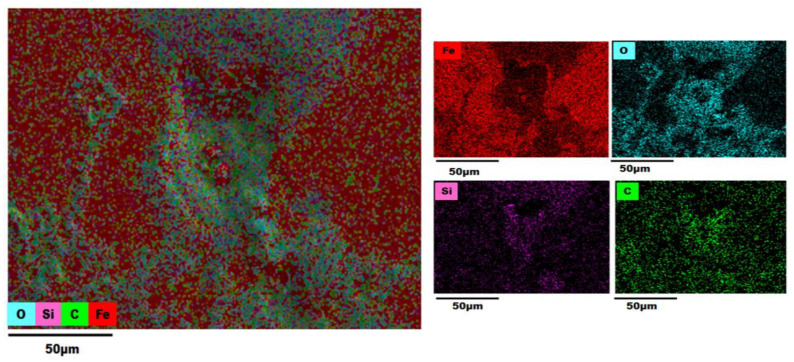
EDS map of a 90° elbow exit surface after the test for 10 wt.% sand fines concentration.

Other observations can be made here. The pits in low particle concentration propagate discretely, while in 5 wt.% sand fines concentration showed a trend of extension in flow direction as shown in Figure 10. The pits in 5 wt.% and 10 wt.% sand fines concentration had a larger size with no-ring corrosion area around the pits as shown in Figure 10 and Figure 11. Though, the pits in 2 wt.% sand fines concentration appeared as smaller shallow pits and encircled by ring areas with corrosion products (Figure 9). After exposure of 90° elbow exit section to a slug flow with the 5 wt.% sand fines concentration, as shown in Figure 10, a clear elongated pitting pattern can be perceived and the corrosion products were shifted at the tip of the pits and not still concentrated around the vicinity of the pit. The elongated pitting corrosion pattern with the erosive mark and corrosion product concentrated inside and outside of the pits can be discerned more clearly from micrographs. With the concentration of the sand fines increased to 10 wt.%, it can be seen from Figure 11, the crack mark over the corrosion area in the slug flow direction. In 5 wt.% sand fines concentration, some cracks can be recognized within some corrosion products deposited at the tip and inside of the pits as shown in Figure 9. The corrosion product is shown in Figure 9, Figure 10 and Figure 11 is proved by Figure 12, Figure 13, Figure 14 and Figure 15.

When the concentration of 10 wt.% sand fines were introduced into the slug flow, the pitting profile transformed from elliptical to undercutting with corrosion products concentrated inside and outside pits as shown in Figure 11. Many deep and narrow pits were perforated on the elbow exit for 10 wt.% sand fines concentration. An intriguing observation from Figure 10 is that most of the pits were contiguously combined to adjacent pits which can be discerned from the microscopic images. Multiple impacts of sand fines at the internal surface of the elbow resulted in cutting and ensue plastic deformation in the downstream section. Furthermore, plastic deformation and metal cutting increases with an escalation of sand fines concentration and incurred strain hardening and makes the internal surface extremely pregnable to corrosion [38].

After erosion-corrosion of the elbow in high sand fines concentration slug flow, the pits gravitate to enlarge in flow direction and coalesce with adjacent perforation sites in their environs. The growth of coalescence perforation sites generates wide and irregular morphology on the material surface. For high sand fines concentration, mesh type pitting corrosion propagates on the surface and eventually create interconnected corrosion pits in the flow direction. At 5 wt.% sand fines concentration, new pitting initiates and propagates individually in the flow direction, and eventually forms a stable pit. Particularly in 10 wt.% sand fines concentration, there were shallow depressions of mesh type structure grew on the surfaces after 10 h of slug exposure as illustrated in Figure 11. This type of pits propagation had become more evident and more severe at high sand fines concentration flow conditions, including the pits being larger. By visualizing the surface morphology, it can be concluded that erosive wear mechanisms depend on impact angles and particle-particle interaction and varied significantly with the change of sand fines concentration.

### 3.3. EDS (Energy-Dispersive X-ray Spectroscopy) Analysis

An EDS analysis of the AISI 1018 steel 90° elbow exit section was also employed in the examination of elemental distribution and mapping before and after the test as shown in Figure 12, Figure 13, Figure 14 and Figure 15.

The color-coded map from EDS analysis reveals the preeminence of iron atoms vicinity the pit perforation sites after the test. Also, it can be explicated that the increase in the oxygen atom distribution signifies the containment of iron oxide deposits that increases localized corrosion inside the perforation sites. In addition, the presence of Si outside and inside pit as shown in Figure 13, Figure 14 and Figure 15 confirmed that the fine sand particles were embedded on the surface after the test.

The electron micrographs of the corrosion products inside and outside pits for the 2, 5, and 10 wt.% sand fines concentration was shown in the preceding section. For 10 wt.% sand fines concentration, the corrosion layer had a perceptible mesh structure; with the scale deposit develops in a flow direction. There was minimal pitting corrosion on the elbow specimen surface for 2 wt.% sand fines concentration relative to 5 wt.% and 10 wt.%.

It can be observed from EDX analysis (EDX spectrum before and after tests are reported in Figures S1–S4 (Supplementary Materials)) that after the test the distribution of iron atoms reduced inside the perforation sites while an upsurge of the oxygen atoms found inside the pits with considerable inflation in the carbon level around the perforation sites. Concurrently, the weight percentage of iron was greatly reduced for high sand fines concentration flow conditions, designating that sand fines in the carrier phase effectuate strong corrosion of the carbon steel material. Additionally, the weight percentage of iron after the test is high for low fine sand concentration in carrier phases, disseminating that the corrosion level is minor. EDX analysis elucidates that the weight percentage of oxygen and carbon atoms were significantly higher after the test as shown in Table 3. Hence, the results reveal that the corrosion products are contrived of Fe_2_O_3_. A comparison of Figure 13, Figure 14 and Figure 15 show that 10 wt.% sand fines concentration in slug flow induces severe pitting corrosion, which was also found on the surface. The results proved that the increasing sand fines concentration significantly reduced iron atoms, analyzed at two different spots marked as 1 (inside pit) and 2 (outside pit). Table 3 indicated that the worn surface consisted of an iron atom between 89.8% and 49.1% in 2 wt.% sand fines impact conditions. In comparison with 5 wt.% sand fines impact conditions, the amount of iron has been reduced between 49.6% and 25.4%, the deduction obtained in the iron atom specified that the erosion enhanced metal corrosion. Yang and Cheng [39] also obtained the same conclusion that the corrosion reaction of carbon steel ameliorates with the sand loading in the carrier phase.

### 3.4. Mass Loss

**Figure 16:**
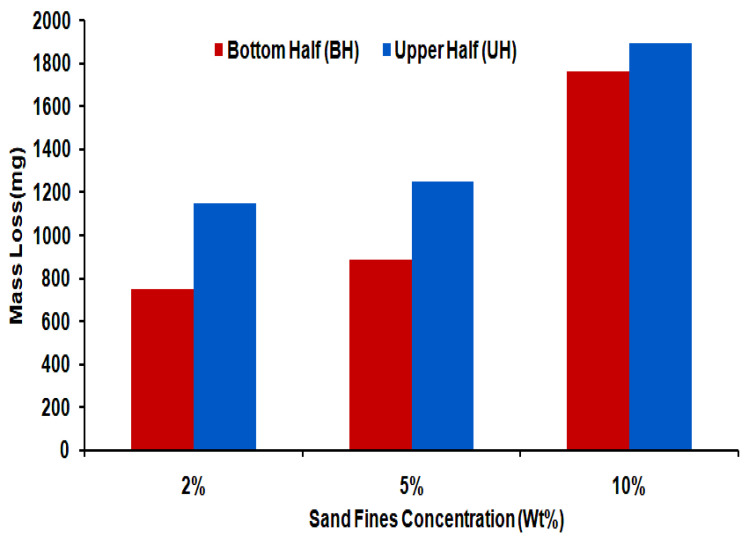
Mass loss in 1018 CS bottom half and upper half elbows section after exposure of slug flow with different sand fines concentration for 10 h.

As illustrated in Figure 16, the mass-loss rate in the upper half section is approximately 1.07 to 1.53 times higher relative to the bottom half section for all evaluated cases. The erosion damage in 10 wt.% sand fines concentration is more serious than low sand fines concentration. This deviation in the mass-loss rate in the upper half section can also be explained by the high number of particles in the gas phase lead to an outrageous degradation rate [14]. Therefore, the erosion-corrosion of the upper half elbow section should be emphasized more in slug flow.

In all cases examined, the mass loss rate in carbon steel 90° elbow with 10 wt.% sand fines concentration was 1.7 times higher than 5 wt.% and 1.92 times higher in comparison to 2 wt.% sand fines concentration in the erosive slug flow as shown in Table 4. The difference is very significant for the sand fines concentration with 2 wt.% loading, where an increase in the erosion-corrosion rate of 93% can be noticed with the change in 10 wt.% sand fines concentration. In horizontally oriented elbow pipe, the liquid film and turbulent slug body flow continually, the centrifugal force accelerates the liquid to the top of the bend section. The erodent is mainly transported by liquid phase and sand fines in the liquid film incurred erosive wear at the top pipe section and consistent with the findings of Peng et al. [40].

## 4. Conclusions

In this work, an experimental investigation was employed to evaluate the erosion-corrosion in 90° elbows under erosive slug flow conditions with 2, 5, and 10 wt.% sand fines concentration. The investigation was performed for the horizontally oriented AISI 1018 steel elbows installed in the multiphase flow loop where the water and air flow to simulate slug flow conditions. The main conclusions drawn from this experimental investigation are:

1. The erosion-corrosion mechanism diversified substantially with the increase in sand fines concentration in slug flow. From mass loss rate measurements, erosion-corrosion of AISI 1018 CS in 10 wt.% sand fines concentration increased up to 93% relative to the 2 wt.% sand fines concentration for slug flow conditions.
2. The microscopic images and surface topography of elbow exit surfaces reveal that impact of sand fines leads to the development of circular, elongated, and coalescence under slug flow conditions. In addition, plastic deformation and metal cutting increases with an increase of sand fines concentration and incurred strain hardening and make the internal surface extremely susceptible to corrosion.
3. For 2 wt.% and 5 wt.% sand fines concentration in slug flow, the maximum particle wall impaction always occurs on the outer arch of the elbow between 60 and 90 degrees downstream adjacent to the outlet. Furthermore, examining 10 wt.% sand fines concentration showed erosion hotspot extended between 30 and 90 degrees downstream as the concentration increases.
4. Under horizontal erosive slug flow conditions, an increase in sand fines concentration significantly increased degradation rate in the upper half section, the maximum erosion was found in the top of the elbow for all levels of concentration in the carrier phase. Additionally, the surface morphologies and EDS show that there are corrosion products around and inside the pitting holes perforated on the surface of the elbow after the test.

The present study congregates quantitative and qualitative experimental procedures to evaluate a 90° elbow erosive wear mechanism and provides significant information on the issue of erosion-corrosion due to sand fines. The results indicate that the sand fines concentration level in the carrier phase is a crucial factor in the mitigation of erosion-corrosion damage in the process where sand fines are present in the system.

## Figures and Tables

**Table 1:** Composition elbow material (wt.%).

1018 CS								
Si	Cr	Cu	P	C	S	Ni	Mn	Fe
0.26	0.21	0.25	0.045	0.2	0.035	0.3	0.52	98.18

**Table 2:** Composition of silica sand (wt.%).

SiO_2_	Al_2_O_3_	Fe_2_O_3_	Na_2_O	MgO	CaO
98.08	1.17	0.28	0.03	0.22	0.22

**Table 3:** Weight percentages obtained by EDS.

Element	Before Test	Pit A		Pit B		Pit C	
		1	2	1	2	1	2
	2 wt.% sand fines concentration						
C	7.6	16.3	7.1	22.7	13.4	12.7	11.2
O	-	15.8	2.7	23.1	6.7	31.7	20.8
Fe	92.4	64.5	89.8	53.5	79.1	49.1	67.6
Si	-	1.8	0.4	0.2	0.3	3.9	0.3
	5 wt.% sand fines concentration						
C	7.6	27.8	11.8	19.1	11.0	18.1	12.7
O	-	19.5	37.4	37.6	34.6	28.3	31.7
Fe	92.4	49.6	43.0	25.4	47.2	46.4	49.1
Si	-	1.8	4.1	15.4	4.1	3.5	3.9
	10 wt.% sand fines concentration						
C	7.6	22.0	12.7	35.5	10.3	23.0	10.3
O	-	29.2	31.7	23.9	31.5	28.8	33.9
Fe	92.4	41.1	49.1	36.7	51.5	42.0	47.5
Si	-	4.0	3.9	2.1	3.9	2.4	4.1

**Table 4:** Loss rates of tested 90° elbows for slug flow.

Material	*V*_SG_ (m/s)	*V*_SL_ (m/s)	Flow Time (h)	Particle Size (µm)	Particle Concentration (wt.%)	Mass Loss Rate (kg/m^2^·s)
1018 CS	2.5	0.5	10	50 ± 2	2	2.77 × 10^−6^
1018 CS	2.5	0.5	10	50 ± 2	5	3.12 × 10^−6^
1018 CS	2.5	0.5	10	50 ± 2	10	5.33 × 10^−6^

## Supplementary Materials

The following are available online at https://www.mdpi.com/1996-1944/13/20/4601/s1, Figure S1: Elemental phase spectra before the test for 1018 CS, Figure S2: Elemental phase spectra after the test for 1018 CS with 2 wt.% sand fines concentration, Figure S3: Elemental phase spectra after the test for 1018 CS with 5 wt.% sand fines concentration, Figure S4: Elemental phase spectra after the test for 1018 CS with 10 wt.% sand fines concentration.
