# Supplementary material for: Effect of Sand Fines Concentration on the Erosion-Corrosion Mechanism of Carbon Steel 90° Elbow Pipe in Slug Flow
Source: Materials (Basel). 2020 Oct 16;13(20):4601. doi: 10.3390/ma13204601 (PMC7602876; doi:10.3390/ma13204601)
Supplement: Supplementary file 1 [file materials-13-04601-s001.pdf]

materials

MDPI

Supplementary Materials.

Effect of Sand Fines Concentration on the Erosion-Corrosion Mechanism of Carbon Steel 90° Elbow Pipe in Slug Flow

1. Energy-dispersive X-ray spectroscopy (EDX) before and after the test for Slug Flow

The EDX spectrum obtained before and after the test to understand the change in chemical composition.

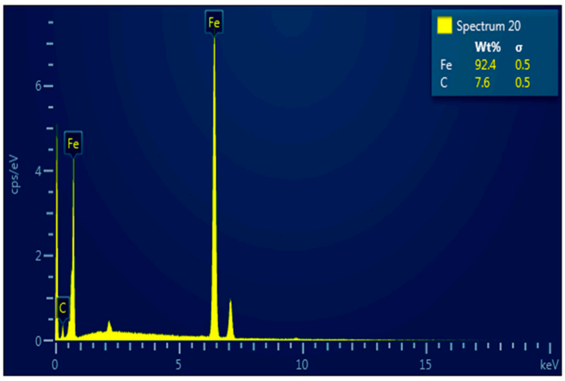

Figure S1. Elemental phase spectra before the test for 1018 CS

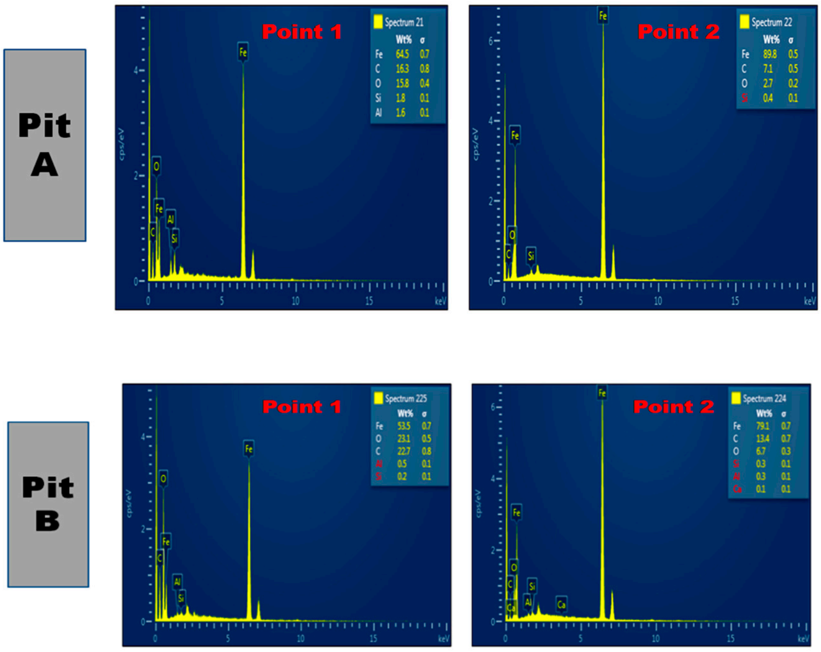

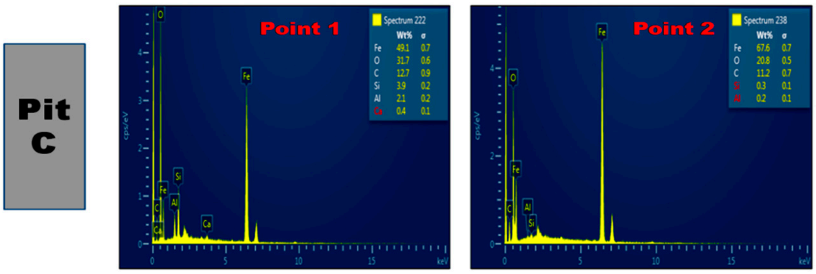

Figure S2. Elemental phase spectra after the test for 1018 CS with 2 wt.% sand fines concentration

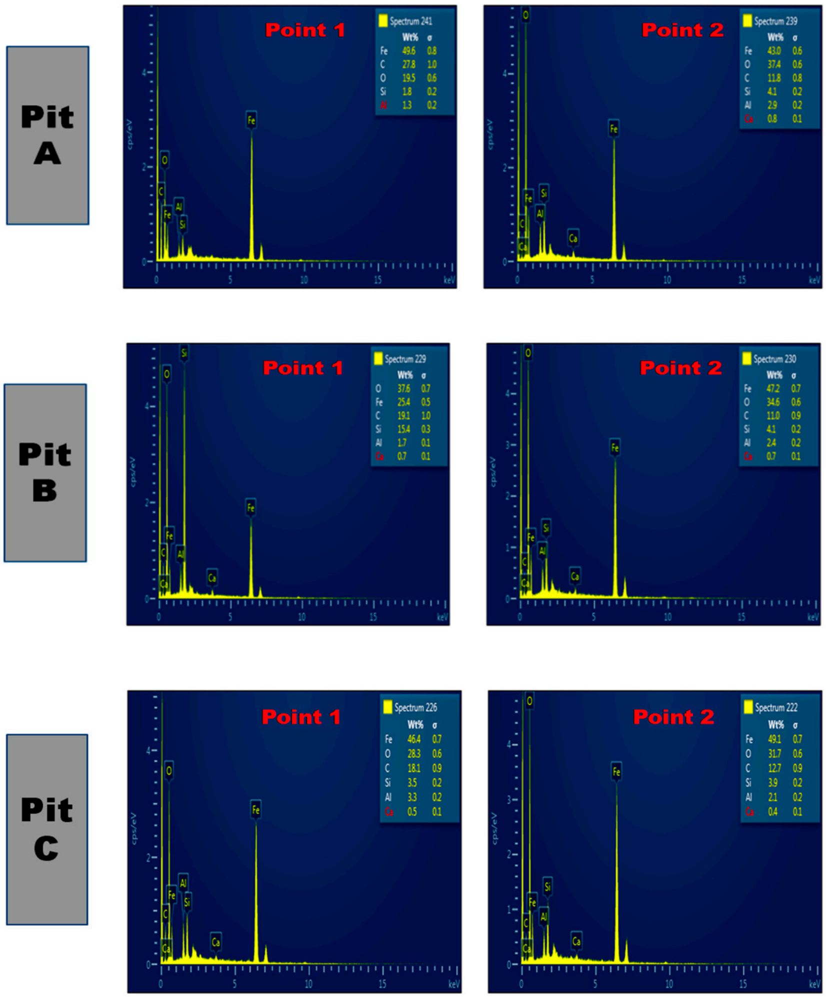

Figure S3. Elemental phase spectra after the test for 1018 CS with 5 wt.% sand fines concentration

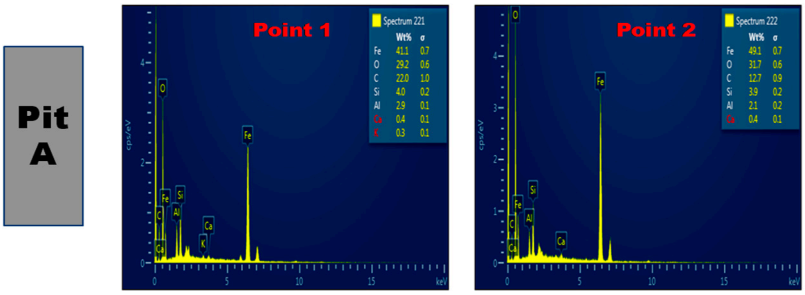

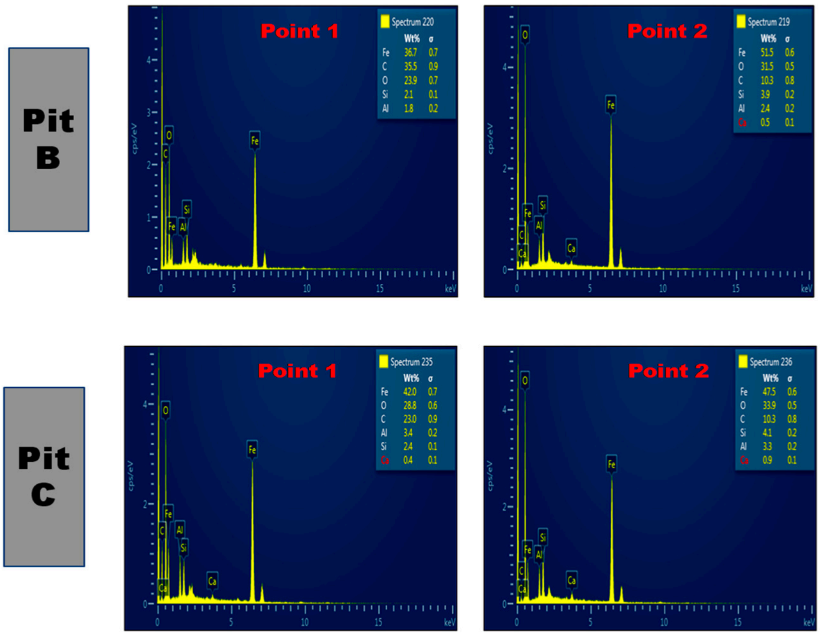

Figure S4. Elemental phase spectra after the test for 1018 CS with 10 wt.% sand fines concentration

© 2019 by the authors. Submitted for possible open access publication under the terms and conditions of the Creative Commons Attribution (CC BY) license (http://creativecommons.org/licenses/by/4.0/).
